# The Demographic Specific Abdominal Fat Composition and Distribution Trends in US Adults from 2011 to 2018

**DOI:** 10.3390/ijerph191912103

**Published:** 2022-09-24

**Authors:** Furong Xu, Jacob E. Earp, Bryan J. Blissmer, Ingrid E. Lofgren, Matthew J. Delmonico, Geoffrey W. Greene

**Affiliations:** 1 School of Education, University of Rhode Island, Kingston, RI 02881, USA; 2 Department of Kinesiology, University of Connecticut, Storrs, CT 06269, USA; 3 Department of Kinesiology, University of Rhode Island, Kingston, RI 02881, USA; 4 Department of Nutrition and Food Sciences, University of Rhode Island, Kingston, RI 02881, USA

**Keywords:** visceral adipose tissue area, subcutaneous adipose area, visceral to subcutaneous adipose area ratio, adults, health disparities

## Abstract

Despite the rising awareness of abdominal adiposity associated health problems and demographic health disparities, research is lacking about abdominal fat trends using a national representative sample of US adults. Our purpose was to examine national demographic specific abdominal fat composition and distribution trends from 2011 to 2018. This trend analysis was using National Health and Nutrition Examination Survey data (*n* = 13,163). Visceral adipose percent (VAT%), visceral adipose tissue area (VAA) and visceral to subcutaneous adipose area ratio (VSR) were utilized in data analyses. Multiple polynomial linear regression was utilized with adjustment for confounding variables. Our findings revealed that VAT%, VAA and VSR trends were concave among all demographic groups. The VAT%, VAA and/or VSR changes were observed in most demographic groups (*p* < 0.05) except younger, White and Black respondents. The pattern was consistent with biennial increases up to 2014 or 2016 followed by decreases in 2017–2018. There were demographic disparities, with middle-aged respondents and Hispanics having the most evident VAT%, VSR and/or VAA changes biennially when compared to their counterparts (*p* < 0.05). In conclusion, abdominal fat composition and distribution increased before 2014 or 2016 but decreased afterwards with variations by age and/or race/ethnicity. Further research is needed to explore the possible causes of abdominal fat changes overtime.

## 1. Introduction

The overweight and obesity epidemic has long been a major health problem for US adults given the multitude of health risks associated with storing excess body fat [1]. However, recent research has emphasized regional body fat depots and distribution as an independent risk factor for many obesity-related illnesses [2,3,4,5,6,7,8,9,10,11,12,13,14,15]. More specifically, researchers have reported that visceral fat depots are associated with multiple chronic inflammatory diseases (e.g., type II diabetes, cardiovascular disease), metabolic syndrome, and all-cause mortality [4,5,6,7]. Additionally, studies also indicated that visceral adipose tissue area (VAA) and visceral to subcutaneous adipose area ratio (VSR) were also important for health [3,8,9,10,11,12,13,14,15]. Some research found the association between multiple metabolic risk factors and VAA [8,9,10] and VSR [3,11,13] that is independent of those seen with body mass index or overall body fat. Other researchers identified a connection between VSR and cardiovascular risks [14] or indicated that VSR was associated with type II diabetes [15]. These studies have concluded that visceral fat that is stored inside the abdominal cavity and around the internal organs carries with it much greater health risks than the subcutaneous fat that makes up most of the fat stored by the body [2,3,4,5,6,7,8,9,10,11,12,13,14,15]. All in all, abdominal fat-related measures: visceral fat, VAA and VSR have become variables of interest for better understanding the health risks of obesity-related illnesses [3,4,5,6,7,8,9,10,11,12,13,14,15]. Although the prevalence and trend (changes over time) of overweight or obesity in adults have been well documented [1], the trend of abdominal fat composition and distribution (e.g., VAA, VSR) is lacking especially using precisely measured visceral fat, VAA, and VSR. Since these abdominal fat measures are associated with cardiometabolic diseases [3,4,5,6,7,8,9,10,11,12,13,14,15], it is crucial to understand their changes over time in US adults. This will allow health practitioners to assess and evaluate nationwide abdominal fat progress and propose appropriate prevention and intervention strategies to address abdominal obesity to ameliorate its related chronic conditions and improve public health.

Previous research indicated that visceral adipose tissue varied by age, sex, race/ethnicity, income [16,17,18,19,20]. Specifically, men have higher VAA than women [16], Black men have less visceral fat than their White counterpart [16,17,18], and both education income levels are inversely related to visceral fat depots [19,20]. Moreover, a recent cardiometabolic health trend study revealed that US adults’ cardiometabolic health has been worsening from 1999 to 2018 and there are cardiometabolic health disparities by sex, age, race/ethnicity, education and family income [21]. Given the visceral adipose tissue variation by demographics [16,17,18,19,20] and its related cardiometabolic health trend [3,4,8,9,10,11,12,13], it is important to understand abdominal adiposity trends by demographics. Available studies on abdominal adiposity trends using anthropometric index—waist circumference—revealed that there were race/ethnic disparities [22,23]. Specifically, there have been annual waist circumference increases were observed among people with higher family income, in White, Black and Mexican Americans. However, Mexican Americans had the highest annual increase rate [22]. Another study found increases in Hispanic, White, Asian but not in Black Americans [23]. Given the inconsistent findings on abdominal adiposity trends when assessed via waist circumference [22,23], there is a need to extend previous research to examine demographic specific abdominal fat composition and distribution trends. To the best of our knowledge, there are no such trend studies available in the existing literature using precisely measured abdominal fat composition and distribution using dual-energy X-ray absorptiometry (DXA). We believe that such information is essential as it provides a dynamic view of accurately measured abdominal fat composition and distribution in the US adult population. This allows for more precise measurement of visceral fat, which provide a greater link to cardiometabolic health than measures such as body mass index. Such information can be utilized by public health professionals to assist in the development of abdominal fat-related health needs assessments. Researchers in public health can use the information to generate hypotheses for further research to address healthy fat distribution. Accordingly, the present study aimed to examine the abdominal fat composition and distribution trends by demographics using a nationally representative adult sample.

## 2. Method

The present study is a trend analysis utilizing National Health and Nutrition Examination Survey (NHANES) dataset from 2011 to 2018 (four data cycles) [24]. The age range for the present study is 18–59 years since DXA scans were only administered to participants up to age 59 years [24]. In total, 16,142 out of 39,156 respondents were identified for the present study. Identified participants were further excluded if they missed total body mass, visceral adipose tissue mass, VAA, and subcutaneous fat area data (*n* = 2979). Ultimately, a total of 13,163 participants were used for analysis. This study has been approved by the Institutional Review Board (IRB) at the University of Rhode Island (IRB#1935395–1).

### 2.1. Abdominal Fat Composition and Distribution

Abdominal adiposity-related measures were obtained from the NHANES’s DXA (Hologic, Inc., Bedford, MA, USA) scans. From these scans, three distinct variables were reported. Firstly, visceral adipose tissue percentage (VAT%) provided a measure of visceral adipose tissue mass relative to total body mass, Secondly, VAA provided an absolute measure of visceral adipose deposits (cm^2^) which stored inside the abdominal cavity [24,25]. Third, the VSR was used to describe the relative distribution of adipose between visceral adipose to subcutaneous components [11,12].

### 2.2. Confounding Variables

Demographics were retrieved from the NHANES dataset and included sex, age, race/ethnicity, education and the ratio of family income to the poverty level [24]. Categories for each of these demographic variables were collapsed into fewer categories as done previously to minimize data fragmentation [26]. The specific changes were (1) age was categorized into 18–39 years and 40–59 years; (2) race/ethnicity was categorized into White, Black, Hispanic (including Mexican American and other Hispanic), and Other; (3) education was categorized as higher school or less (including less than 9th grade, 9–11th grade, high school graduate/GED or equivalent), or some college or more (including some college or AA degree, college graduate or above). (4) Additionally, ratio of family income to poverty (0–4.99, 5 or above) has been classified into two categories: at or above (≥1), below poverty level (<1) [24,26]. In addition to demographics, body fat percentage has been included as one of confounding variables for certain analyses due to its possible influence on study variables [27,28].

### 2.3. Data Analysis

The combined 8-year mobile examination center weights were used as the sample weight for all the analyses: calculating the estimate and its standard error; design-based methods were also used to estimate the standard error based on NHANES analysis guidelines [29]. The descriptive results for demographic characteristics were presented as weighted mean ± standard errors or *n* (weighted%), and comparisons (*p*-values) were done by linear or logistic regression model. VAT%, VSR and VAA at each time point were presented as mean (95% confidence interval) and *p* values was obtained by univariate polynomial linear regression model. Furthermore, according to preliminary visualization of the figures for VAT%, VSR and VAA trends over study period 2011–2018, we tested the linear and non-linear (quadratic) trends using polynomial linear regression model using the mean of VAT%, VSR and VAA. The *p* values for trends were estimated from multiple polynomial linear regression in which the data cycle was treated as continuous variable, adjusted for age, sex, race/ethnicity, education, and ratio of family income to the poverty level. The analysis for VSR and VAA also adjusted for body fat percentage. Polynormal linear regression: y = β_0_ + β_1_ × time + β_2_ × time^2^ + β_i_ × (adjusted variables) in which β_1_ gives the rate of change over time; the coefficient β_2_ tells both the direction and steepness of the curvature (a positive value indicates the curvature is upwards while a negative value indicates the curvature is downwards). Furthermore, the interaction terms stratified variables x cycle (linear and quadratic) was added into the model to examine the effect of the interaction between stratified variables and cycle to investigate whether there are changes over four data cycles (2011–2018) in mean differed between the stratified variables. All the analysis was conducted using Statistical Analysis Software (SAS) 9.4 (SAS Institute Inc., Cary, NC, USA) with a *p* value less than 0.05 as the chosen significant level.

## 3. Results

**Figure 1:**
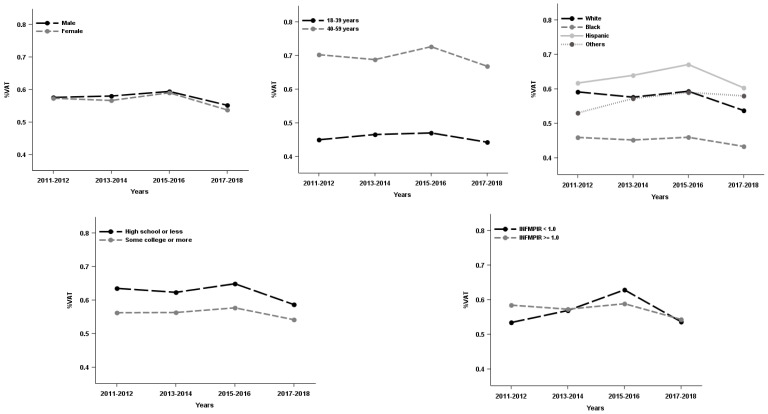
The mass of visceral adipose tissue relative to total body mass (VAT%) trends in US adults by demographics, National Health and Nutrition Examination Survey 2011–2018. INFMPIR = the ratio of family income to the poverty level.

**Figure 2:**
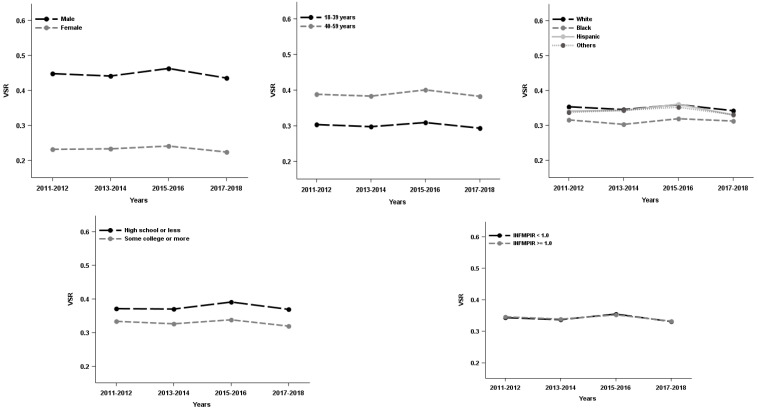
The visceral to subcutaneous adipose area ratio (VSR) trends in US adults by demographics, National Health and Nutrition Examination Survey 2011–2018. INFMPIR = the ratio of family income to the poverty level.

**Figure 3:**
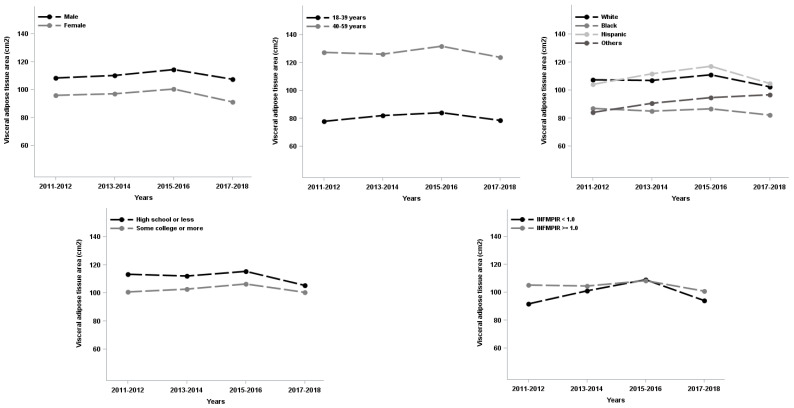
The visceral adipose tissue area trends in US adults by demographics, National Health and Nutrition Examination Survey 2011–2018. INFMPIR = the ratio of family income to the poverty level.

The study sample were approximately half females (48.6%), half middle age or older (49.0%, 40–59 years). About one third were minorities (38.8%) and had high school or less in education (34.8%), and less than one fifth of respondents (16.6%) had family income below the poverty level. Additionally, about one third of respondents were categorized as overweight (31.2%) and slightly more than one third as obese (37.4%). Males had higher VSR and VAA than females whereas females had higher subcutaneous fat area than males (see Table 1). Moreover, VAT%, VSR or VAA prevalence was varied by sex, age, race/ethnicity, education, and family income. Females, the younger group (18–39 years), Black, and people with higher education tended to have lower VAT%, VSR and VAA (see Table 2). Over the study period (2011–2018), VAT%, VSR and VAA increased for Hispanic and/or respondents who had family income below the poverty level up to 2016, and then decreased afterwards (see Figure 1, Figure 2 and Figure 3).

The adjusted demographic specific abdominal composition trend analyses revealed the non-linear VAT% change or the concave trends for VAT% overtime in males, middle-aged respondents, Hispanic, and respondents with lower education level and family income, and the curvilinear model was positive to negative slope. The trend for VAT% is concave overtime; values increased and then decreased over time with peak values reported around 2014 or 2016. Specifically, in males, the VAT% was increased by 0.017% [β1 × (2 − 1) + β2 × (2^2^ − 1^2^) = 0.050 − 0.011 × 3 = 0.017] between 2011–2014, but after 2014, the VAT% was decreased by 0.005% [β1 × (3 − 2) + β2 × (3^2^ − 2^2^) = 0.050 − 0.011 × 5 = −0.005] to 0.027% [β1 × (4 − 3) + β2 × (4^2^ − 3^2^) = 0.050 − 0.011 × 7 = −0.027), the slope thus becomes negative (See Table 3). A similar pattern was observed in middle aged respondents (aged 40–59 years), Hispanic, respondents with high school or lower education and family income below poverty level (see Table 3). There was a VAT% change rate difference between racial/ethnic groups, but no difference observed in other demographic groups (male vs. female, younger vs. older, lower vs. higher education, lower vs. higher family income) (see Table 3).

The adjusted demographic specific abdominal distribution trends also are concave for both VSR and VAA over the study period. Specifically, the adjusted analyses revealed that among males, the linear parameter increased (*p* = 0.005) at first whereas the quadratic parameter decreased (*p* = 0.008) afterwards. That is, the VSR was increased by 0.015 (0.039–0.008 × 3) from 2011 to 2014, however, VSR decreased biennially by 0.001 (0.0039–0.008 × 5) to 0.017 (0.039–0.008 × 7), respectively from 2015–2016 to 2017–2018. A similar pattern observed in respondents who are aged 40–59 years, Hispanic, respondents with lower education and the ratio of family income to the poverty level below federal poverty level (see Table 3). There were VSR biennial change rate difference by race/ethnicity but not in other demographic groups (see Table 3). For VAA, the results were relatively consistent with the findings for VSR except there was also VAA biennial change rate difference by age (see Table 3).

## 4. Discussion

To the best of our knowledge, the present study is the first to examine the national demographic specific trends on abdominal fat composition and distribution using precisely measured visceral fat via DXA. The findings from the present study indicated that the trends were concave for both abdominal composition (VAT%) and distribution (VSR and VAA) and were not linear. There were abdominal composition and distribution disparities by race/ethnicity and/or age. Most notably, VAT%, VSR, and VAA changes were greatest in Hispanics and/or middle-aged respondents (VAA only) even after accounting for total adiposity.

The findings from the present study indicate that the demographic specific abdominal composition (VAT%) trends were concave and VAT% increased across the board over the first of 2–3 data cycles up to 2014 or 2016 but the increase was not constant. Previous studies on abdominal adiposity trend using anthropometric index-waist circumference [22,23,30] reported the waist circumference increased steadily in US adults from 1999 to 2012, or 1999 to 2016 or 2011 to 2018, respectively [22,23,30]. Although waist circumference is widely accepted anthropometric index for abdominal visceral fat [23,31,32], the direct measure of visceral fat is more accurate. Thus, the present study adds to the existing literature by examining the demographic specific abdominal fat composition trends using direct and precisely measured visceral adipose tissue mass in relevant to total body mass–VAT% which is an important risk factor for cardiovascular and metabolic diseases [4,5,6,7].

Our findings indicated that VAT% increase in males, middle aged respondents (40–59 years old), Hispanic, respondents with lower education, or family income below the federal poverty line from 2011 to 2014 or 2011 to 2016, then there were decreases after 2014 or 2016, respectively. Previous studies using waist circumference as a proxy for abdominal adiposity found the waist circumference increased in both sexes, White, Black and Mexican American [22,30], whereas Liu colleagues (2021) reported waist circumference increase in White and Hispanic samples only [23]. Their findings are somewhat consistent with ours but we only found statistically significant VAT% increase in Hispanics from 2011 to 2016 then decreases afterward. Additionally, the greatest biennial change observed in Hispanics versus other racial/ethnic groups was consistent with Wang and colleagues’ finding as well [22]. Of course, direct comparison is not possible due to different study outcomes (VAT% vs. waist circumference) and different confounding variables adjusted for analysis.

Another point to consider when comparing differences between groups in the increases over time between demographic groups, is that the observed trends maybe partially explained by differences in starting point of these groups. For instance, as middle aged (40–59 years old) adults have higher visceral adiposity than younger (18–39 years old), societal changes that effected both younger and older adults may therefore have a great effect on middle aged adults [33] simply because there is more room for improvement. It is also worth noting that there was a VAT% trend disparity by racial/ethnic groups and VAT% was constantly higher in Hispanic in comparison to other racial/ethnic groups at all time points. However, no trend disparities were observed in other demographic groups even though VAT% was higher in respondents with lower education in comparison to their counterparts at all data points which is consistent with existing literature [19].

To the best of our knowledge, the present study also is the first study examining the demographic specific abdominal fat distribution trends using VSR and VAA which are related to cardiometabolic diseases [3,11,12,13,14,15]. We note that VSR and VAA increased among Hispanics, less educated and/or respondents with lower family income around 2013–2014 or 2015–2016, then VSR or VAA were gradually decreased afterwards. Additionally, our findings indicated that respondents who were better educated and/or had higher family income also increased their VSR and VAA before 2014 but decreased afterwards. Moreover, our findings revealed that there were VSR and VAA trend disparities by race/ethnicity and/or age from 2011 to 2018. All in all, it is still a surprise for us to see this while the obesity epidemic was worsening over time [1,23,34], body mass index increased significantly from 28.7 kg/m^2^ to 29.8 kg/m^2^ between 2011 and 2018 [23], and the percent body fat trend from 2011 to 2018 was relative stable overall and among different racial/ethnic groups [23]. However, our findings are independent of body fat as our trend analyses adjusted for body fat percentage. Clearly, the VSR and VAA were changed overtime, but its changes were different from the change in body mass index or percent body fat that was expected [23,34].

Our findings raise more questions than answers, but they indicate that something positive could have happened between 2013–2014 and 2015–2016. While a direct explanation of these trends is beyond the scope of the present study, we suggest future research should investigate societal changes that occurred during these inflection periods. Furthermore, their results could be influenced by a range of different factors such as changes in lifestyle trends, cultural movements, economic trends, and federal policy changes. For example, one of our previous studies found that improving dietary quality improved fat distribution when accounting for other factors like sex, race/ethnicity, education and family income [28]. Given national trends in adiposity [23], it should not be surprising that there are initial increases in visceral adipose measures across most groups. However, a more surprising result is the apparent decreases in visceral fat deposits following 2016 found in the present analyses. Some noteworthy changes during this time would be enactment of the 2015–2020 Dietary Guidelines for Americans, a continued increase in the number of US adults meeting the physical activity recommendations [35], a stark decrease in national poverty rate after 14 years of increases [36]. It could also possibly be related to the passage of the Affordable Care Act [37,38]. Looking at the timeline of the Affordable Care Act, it was passed in 2010, but enrollment did not begin until October 2013 and it was not open to everyone until 2014 [38]. For context, Affordable Care Act increased the percentage of the population with health care coverage, primarily by mandating coverage for those without coverage (who were primarily lower income, less educated, ethnic minorities) and expanding Medicaid to cover more low-income Americans. Additionally, the percent of uninsured from 2013 and 2019 declined more for Hispanics than any other racial/ethnic groups [37]. All of these factors (dietary quality, physical activity rates, poverty levels, and access to health coverage) have all been independently linked with health outcomes and it is quite possible that these health improvements would also show up in visceral adipose levels. This is an area worthy of further inquiry as it may provide guideline for best practices, policies, and interventions to improve cardiometabolic health.

The cross-sectional nature of the data used for this study precludes any argument of causality; nevertheless, our findings suggest that certain groups might be more vulnerable (e.g., less educated, Hispanic), which needs to be considered while addressing abdominal fat distribution-related health issues in adults for public health. Regardless, what has been implemented in the US between 2011 and 2018 for public health promotion seems to be working in reducing abdominal fat composition and distribution. We view this as very encouraging news for public health practitioners. While the biennial change rates for VAT%, VSR and VAA were relatively small, they were statistically significant. However, we do not know whether this trend continued after 2018; therefore, it cannot be determined if the reduction reached clinically significant levels. Further research is needed to investigate beyond 2018 to see whether abdominal fat distribution continued to improve or worsen and explore the causes or phenomenon that could possibly influence abdominal fat distribution changes overtime.

### Strength and Limitations

A major strength of the present study is the first study to examine national abdominal fat composition and distribution trends using VAT%, VSR, VAA which were derived from body composition values (e.g., visceral adipose) precisely measured using DXA [24,25,39]. We also controlled for overall body fat percentage to isolate the trends among visceral adiposity. The abdominal fat measures used (visceral adiposity tissue, VSR and VAA) have identified connections with cardiometabolic risk factors [3,4,5,6,7,8,9,10,11,12,13,14,15]. However, limitations of our study include that (1) we were only able to have respondents 59 years or younger since this is a secondary data analysis and is limited to what data are available; (2) causal relationships cannot be determined due to the cross-sectional nature of the data used; (3) we might overlook some confounding variables which might potentially influence our study outcomes.

## 5. Conclusions

The abdominal fat composition and distribution trends from 2011 to 2018 were concave but not linear. For abdominal fat composition trend, biennial change for VAT% was observed in males, middle-aged, Hispanic and respondents with lower education and family income below the federal poverty level, and their VAT% was increased in the first 2–3 data cycles then decreased afterwards. For abdominal fat distribution trends, biennial changes for VSR and VAA were observed in most demographic groups except younger, White and Black groups, and again the VSR and/or VAA increased at first then decreased after 2014 or 2016. In addition, there were abdominal fat composition and distribution trend disparities by race/ethnicity and/or age. The findings are not consistent with trends in body mass index or body fat percentage over this period as reported in previous studies. Further research is needed to examine abdominal fat composition and distribution trends after 2018 to see whether this decrease was sustained, and to examine possible societal changes that could influence the national trends.

## Figures and Tables

**Table 1:** Demographics stratified by sex, NHANES 2011–2018.

Variables	Total	Male	Female	*p*-Values
	*n* = 13,163	*n* = 6638 (51.4%)	*n* = 6525 (48.6%)	
Age, *n* (weighted %)				
18–39 yrs	6996 (51.0)	3657 (52.8)	3339 (49.2)	<0.001 *
40–59 yrs	6167 (49.0)	2981 (47.2)	3186 (50.8)	<0.001 *
Race/ethnicity, *n* (weighted %)				
White	4496 (61.2)	2310 (61.2)	2186 (61.3)	0.967
Black	2958 (11.9)	1456 (11.4)	1502 (12.5)	0.004 *
Hispanic	3294 (17.4)	1594 (18.0)	1700 (16.9)	0.014 *
Other	2415 (9.4)	1278 (9.4)	1137 (9.4)	0.925
Education, *n* (weighted %)				
High school or less	4894 (34.8)	2706 (38.8)	2188 (30.7)	<0.001 *
Some college or more	7252 (65.2)	3401 (61.2)	3851 (69.3)	<0.001 *
Ratio of family income to poverty, *n* (weighted %)				
<1.0	2901 (16.6)	1373 (15.3)	1528 (17.9)	<0.001 *
≥1.0	9137 (83.4)	4671 (84.7)	4466 (82.1)	<0.001 *
Body mass index (kg/m^2^)	28.98 ± 0.14	28.73 ± 0.14	29.24 ± 0.18	0.049 *
Weight status, *n* (weighted %)				
Underweight	312 (1.9)	138 (1.6)	174 (2.2)	0.030 *
Normal	3913 (28.9)	1919 (26.1)	1994 (31.8)	<0.001 *
Overweight	3967 (31.2)	2304 (35.6)	1663 (26.5)	<0.001 *
Obese	4897 (37.4)	2239 (36.0)	2658 (38.8)	0.017 *
Body fat percent	32.77 ± 0.15	27.07 ± 0.14	38.58 ± 0.17	<0.001 *
VAT%	0.57 ± 0.01	0.58 ± 0.01	0.57 ± 0.01	0.718
VAA (cm^2^)	103.42 ± 1.20	110.09 ± 1.35	96.25 ± 1.44	<0.001 *
Subcutaneous fat area (cm^2^)	337.20 ± 3.25	276.49 ± 3.34	402.46 ± 4.13	<0.001 *
VSR	0.34 ± 0.00	0.45 ± 0.00	0.23 ± 0.00	<0.001 *

Note: Data are presented as weighted mean ± standard errors or *n* (weighted%), *p*-values were estimated via multiple regression model, NHANES = National Health and Nutrition Examination Survey, VAT% = visceral adipose tissue percentage, VAA = visceral adipose tissue area, VSR = Visceral adipose tissue area /subcutaneous fat area ratio, * Symbol indicates statistical significance.

**Table 2:** The abdominal fat composition and distribution trends, NHANES 2011–2018.

Variable	2011–2012	2013–2014	2015–2016	2017–2018	*p*-Values for Trend	
**VAT%**	***n* = 2932**	***n* = 3389**	***n* = 3007**	***n* = 2312**	**Linear**	**Quadratic**
Overall	0.57 (0.54–0.61)	0.57 (0.56–0.59)	0.59 (0.57–0.61)	0.54 (0.52–0.57)	0.139	0.064
Sex						
Male	0.58 (0.55–0.60)	0.58 (0.56–0.60)	0.59 (0.58–0.61)	0.55 (0.53–0.57)	0.102	0.055
Female	0.57 (0.53–0.62)	0.57 (0.55–0.59)	0.59 (0.56–0.62)	0.54 (0.51–0.57)	0.269	0.16
Age						
18–39 years	0.45 (0.43–0.47)	0.47 (0.45–0.48)	0.47 (0.45–0.49)	0.44 (0.42–0.46)	0.055	0.037 *
40–59 years	0.70 (0.68–0.72)	0.69 (0.67–0.71)	0.73 (0.70–0.75)	0.67 (0.65–0.69)	0.153	0.094
Race/ethnicity						
White	0.59 (0.56–0.63)	0.58 (0.56–0.60)	0.59 (0.57–0.62)	0.54 (0.51–0.57)	0.407	0.21
Black	0.46 (0.43–0.49)	0.45 (0.43–0.47)	0.46 (0.44–0.48)	0.43 (0.41–0.46)	0.617	0.46
Hispanic	0.62 (0.59–0.64)	0.64 (0.61–0.66)	0.67 (0.64–0.70)	0.60 (0.58–0.63)	0.001 *	0.001 *
Other	0.53 (0.50–0.56)	0.57 (0.55–0.59)	0.59 (0.55–0.63)	0.58 (0.55–0.61)	0.057	0.115
Education						
High school or less	0.63 (0.61–0.66)	0.62 (0.61–0.64)	0.65 (0.62–0.67)	0.59 (0.57–0.60)	0.118	0.036 *
Some college or more	0.56 (0.53–0.60)	0.56 (0.54–0.58)	0.58 (0.55–0.60)	0.54 (0.52–0.57)	0.267	0.186
Ratio of family income to poverty						
<1.0	0.53 (0.46–0.61)	0.57 (0.53–0.60)	0.63 (0.59–0.67)	0.54 (0.48–0.59)	0.040 *	0.036 *
≥1.0	0.58 (0.56–0.61)	0.57 (0.56–0.59)	0.59 (0.57–0.61)	0.54 (0.52–0.57)	0.329	0.164
**VSR**	***n* = 3117**	***n* = 3711**	***n* = 3409**	***n* = 2740**		
Overall	0.35 (0.33–0.36)	0.34 (0.33–0.35)	0.35 (0.35–0.36)	0.34 (0.32–0.35)	0.264	0.214
Sex						
Male	0.45 (0.44–0.46)	0.44 (0.43–0.45)	0.46 (0.45–0.47)	0.44 (0.42–0.45)	0.141	0.143
Female	0.23 (0.22–0.25)	0.23 (0.23–0.24)	0.24 (0.23–0.25)	0.22 (0.22–0.23)	0.142	0.085
Age						
18–39 years	0.30 (0.30–0.31)	0.30 (0.29–0.31)	0.31 (0.30–0.32)	0.29 (0.28–0.30)	0.386	0.33
40–59 years	0.39 (0.37–0.40)	0.38 (0.37–0.39)	0.40 (0.39–0.41)	0.38 (0.36–0.40)	0.352	0.361
Race/ethnicity						
White	0.35 (0.34–0.37)	0.35 (0.33–0.36)	0.36 (0.35–0.37)	0.34 (0.32–0.36)	0.628	0.571
Black	0.32 (0.31–0.33)	0.30 (0.29–0.31)	0.32 (0.30–0.34)	0.31 (0.29–0.33)	0.702	0.69
Hispanic	0.34 (0.32–0.36)	0.34 (0.33–0.35)	0.36 (0.35–0.37)	0.33 (0.31–0.35)	0.033 *	0.024 *
Other	0.34 (0.33–0.35)	0.34 (0.33–0.36)	0.35 (0.33–0.38)	0.33 (0.31–0.35)	0.149	0.144
Education						
High school or less	0.37 (0.36–0.38)	0.37 (0.36–0.38)	0.39 (0.38–0.40)	0.37 (0.35–0.39)	0.165	0.216
Some college or more	0.33 (0.32–0.35)	0.33 (0.32–0.34)	0.34 (0.33–0.35)	0.32 (0.30–0.34)	0.495	0.403
Ratio of family income to poverty						
<1.0	0.34 (0.33–0.36)	0.34 (0.32–0.36)	0.35 (0.34–0.37)	0.33 (0.31–0.35)	0.504	0.449
≥1.0	0.35 (0.34–0.36)	0.34 (0.33–0.35)	0.35 (0.34–0.36)	0.33 (0.32–0.35)	0.344	0.268
**VAA (cm ^2^)**	***n* = 3117**	***n* = 3711**	***n* = 3409**	***n* = 2740**		
Overall	102.40 (96.40–108.41)	103.70 (100.77–106.63)	107.47 (103.05–111.89)	99.64 (94.26–105.03)	0.098	0.072
Sex						
Male	108.30 (102.02–114.57)	110.09 (105.87–114.31)	114.31 (109.14–119.48)	107.37 (101.93–112.81)	0.129	0.118
Female	95.85 (89.13–102.57)	96.98 (93.29–100.68)	100.35 (95.25–105.46)	91.10 (83.72–98.49)	0.126	0.093
Age						
18–39 years	77.80 (72.89–82.70)	81.94 (77.81–86.07)	84.01 (79.70–88.33)	78.49 (73.78–83.19)	0.039 *	0.041 *
40–59 years	127.23 (123.34–131.13)	125.99 (121.42–130.55)	131.67 (125.58–137.75)	123.71 (118.28–129.14)	0.234	0.213
Race/ethnicity						
White	107.25 (100.82–113.68)	106.85 (101.95–111.74)	110.87 (105.81–115.92)	102.27 (94.47–110.07)	0.262	0.214
Black	86.85 (80.32–93.38)	84.92 (80.45–89.38)	86.57 (81.80–91.34)	82.08 (76.97–87.20)	0.786	0.637
Hispanic	104.00 (99.18–108.82)	111.61 (105.20–118.01)	116.97 (111.36–122.59)	104.64 (99.52–109.76)	<0.001 *	<0.001 *
Other	83.98 (77.15–90.81)	90.52 (85.52–95.51)	94.55 (87.30–101.80)	96.60 (88.79–104.40)	0.276	0.528
Education						
High school or less	113.19 (107.55–118.84)	111.95 (108.27–115.63)	115.28 (110.20–120.36)	105.25 (101.44–109.07)	0.183	0.078
Some college or more	100.61 (94.93–106.28)	102.61 (98.35–106.88)	106.28 (101.15–111.41)	100.30 (93.02–107.57)	0.156	0.175
Ratio of family income to poverty						
<1.0	91.58 (79.10–104.05)	100.95 (94.03–107.88)	108.92 (103.37–114.48)	93.93 (82.85–105.01)	0.019 *	0.020 *
≥1.0	105.08 (99.66–110.51)	104.41 (100.86–107.95)	108.23 (103.52–112.94)	100.69 (94.44–106.95)	0.247	0.195

Note: Data are reported as mean (95% confidence interval), *p* value was estimated using polynomial regression model, NHANES = National Health and Nutrition Examination Survey, VAT% = visceral adipose tissue percentage, VSR = Visceral adipose tissue area/subcutaneous fat area ratio, VAA = Visceral adipose tissue area. * Symbol indicates statistical significance.

**Table 3:** The abdominal fat composition and distribution biennial change rate difference by demographics, NHANES 2011–2018.

	Adjusted Coefficient β (95%CI, *p* for Trend)					
	VAT % ^@^		VSR ^&^		VAA (cm^2^) ^&^	
	Linear (β1)	Quadratic (β2)	Linear (β1)	Quadratic (β2)	Linear (β1)	Quadratic (β2)
Sex						
Male	0.050 (0.008–0.092, 0.021 *)	−0.011 (−0.019–−0.002, 0.019 *)	0.039 (0.012–0.066, 0.005 *)	−0.008 (−0.014–−0.002, 0.008 *)	8.087 (0.443–15.732, 0.038 *)	−1.789 (−3.338–−0.241, 0.024 *)
Female	0.043 (−0.021–0.107, 0.186)	−0.010 (−0.022–0.003, 0.119)	0.017 (−0.006–0.039, 0.15)	−0.004 (−0.008–0.001, 0.106)	9.567 (0.856–18.278, 0.032 *)	−1.882 (−3.547–−0.216, 0.027 *)
*p* for different time trends between male and female (sex-cycle interactions ^#^)	0.87	0.932	0.55	0.595	0.8	0.973
Age						
18–39 years	0.038 (−0.009–0.085, 0.113)	−0.008 (−0.017–0.001, 0.093)	0.020 (−0.007–0.048, 0.148)	−0.004 (−0.010–0.001, 0.11)	4.196 (−2.676–11.068, 0.227)	−0.798 (−2.171–0.576, 0.25)
40–59 years	0.056 (0.003–0.110, 0.04 *)	−0.013 (−0.024–−0.002, 0.022 *)	0.038 (0.011–0.065, 0.007 *)	−0.008 (−0.014–−0.002, 0.01 *)	14.540 (6.359–22.722, <0.001 *)	−3.081 (−4.785–−1.376, <0.001 *)
*p* for different time trends between age classification (age classification-cycle interactions ^#^)	0.544	0.391	0.272	0.295	0.041*	0.031*
Race/ethnicity						
White	0.034 (−0.023–0.091, 0.237)	−0.009 (−0.020–0.003, 0.14)	0.021 (−0.009–0.051, 0.165)	−0.004 (−0.010–0.002, 0.154)	6.253 (−2.727–15.233, 0.169)	−1.326 (−3.091–0.439, 0.138)
Black	−0.000 (−0.057–0.057, 0.989)	−0.000 (−0.012–0.011, 0.944)	−0.003 (−0.035–0.029, 0.841)	−0.000 (−0.007–0.006, 0.975)	−0.557 (−9.175–8.061, 0.898)	0.071 (−1.657–1.798, 0.935)
Hispanic	0.122 (0.060–0.184, <0.001 *)	−0.024 (−0.037–−0.012, <0.001 *)	0.058 (0.025–0.091, <0.001 *)	−0.012 (−0.019–−0.006, <0.001 *)	22.438 (14.007–30.868, <0.001 *)	−4.505 (−6.242–−2.769, <0.001 *)
Other	0.072 (0.003–0.142, 0.042 *)	−0.012 (−0.026–−0.002, 0.099)	0.051 (0.015–0.088, 0.007 *)	−0.010 (−0.018–−0.002, 0.016 *)	10.858 (−1.101–22.816, 0.074)	−2.105 (−4.543–0.333, 0.089)
*p* for different time trends between race classification (race-cycle interactions ^#^)	0.030 *	0.043 *	0.003 *	0.004 *	0.005 *	0.007 *
Education						
High school or less	0.064 (0.020–0.108, 0.005 *)	−0.014 (−0.023–−0.005, 0.002 *)	0.034 (0.006–0.062, 0.018 *)	−0.006 (−0.012–−0.000, 0.034 *)	10.929 (2.781–19.077, 0.009 *)	−2.279 (−3.855–−0.703, 0.005 *)
Some college or more	0.038 (−0.015–0.091, 0.158)	−0.008 (−0.019–0.002, 0.113)	0.024 (−0.001–0.050, 0.059)	−0.006 (−0.011–−0.001, 0.031 *)	7.864 (0.865–14.863, 0.028 *)	−1.616 (−3.006–−0.227, 0.023 *)
*p* for different time trends between education groups (education-cycle interactions ^#^)	0.247	0.222	0.659	0.97	0.519	0.461
Ratio of family income to poverty (INFMPIR)						
<1.0	0.104 (0.031–0.177, 0.006 *)	−0.020 (−0.034–−0.006, 0.006 *)	0.042 (0.007–0.077, 0.018 *)	−0.008 (−0.014–−0.001, 0.028 *)	13.343 (4.400–22.286, 0.004 *)	−2.470 (−4.200–−0.740, 0.006 *)
≥1.0	0.033 (−0.012–0.078, 0.148)	−0.008 (−0.017–0.001, 0.082)	0.025 (0.002–0.048, 0.036 *)	−0.005 (−0.010–−0.001, 0.026 *)	7.760 (1.031–14.490, 0.025 *)	−1.678 (−2.974–−0.382, 0.012 *)
*p* for different time trends between INFMPIR groups (INFMPIR-cycle interactions ^#^)	0.047 *	0.08	0.46	0.707	0.268	0.409

Note: Data were analyzed using a polynomial regression model adjusted for sex, age, race/ethnicity, education, and ratio of family income to poverty; ^@^ Data were analyzed using polynomial regression model adjusted for sex, age, race/ethnicity, education, ratio of family income to poverty, ^&^ In addition to what specified for ^@^, analysis also adjusted body fat percentage; NHANES = National Health and Nutrition Examination Survey, VAT = visceral adipose tissue, VSR = Visceral adipose tissue area/subcutaneous fat area ratio, VAA = Visceral adipose tissue area, INFMPIR = the ratio of family income to the poverty level; ^#^ The interaction terms stratified variables*cycle (linear and quadratic) was added into the model to examine the effect of the interaction between stratified variables and cycle, * Symbol indicates statistical significance.

## Data Availability

The dataset utilized for the present study was publicly available on Centers for Disease Control website: https://wwwn.cdc.gov/nchs/nhanes/sasviewer.aspx (accessed on 2 November 2021).
